# A digital enzyme-linked immunosorbent assay for ultrasensitive measurement of amyloid-β 1–42 peptide in human plasma with utility for studies of Alzheimer’s disease therapeutics

**DOI:** 10.1186/s13195-016-0225-7

**Published:** 2016-12-15

**Authors:** Linan Song, D. Richard Lachno, David Hanlon, Adam Shepro, Andreas Jeromin, Dipika Gemani, Jayne A. Talbot, Margaret M. Racke, Jeffrey L. Dage, Robert A. Dean

**Affiliations:** 1 Quanterix Corporation, Lexington, MA USA; 2 Eli Lilly and Company, Windlesham, UK; 3 Lilly Research Laboratories, Indianapolis, IN USA

**Keywords:** Digital ELISA, Ultrasensitive, Aβ_1–42_, Plasma, Alzheimer’s disease, Therapeutic

## Abstract

**Background:**

Amyloid-β 1–42 peptide (Aβ_1–42_) is associated with plaque formation in the brain of patients with Alzheimer’s disease (AD). Pharmacodynamic studies of AD therapeutics that lower the concentrations of Aβ_1–42_ in peripheral blood require highly sensitive assays for its measurement. A digital enzyme-linked immunosorbent assay (ELISA) using single molecule array (Simoa) technology has been developed that provides improved sensitivity compared with conventional ELISA methods using the same antibody reagents.

**Methods:**

A sensitive digital ELISA for measurement of Aβ_1–42_ using antibodies 3D6 and 21F12 was developed. Assay performance was evaluated by repeated testing of pooled human plasma and buffer diluent quality control samples to determine relative accuracy, intra- and inter-assay precision, limit of detection (LOD), lower limit of quantification (LLOQ), dilutional linearity, and spike recovery. The optimized assay was used to quantify Aβ_1–42_ in clinical samples from patients treated with the β-site amyloid precursor protein cleaving enzyme 1 inhibitor LY2886721.

**Results:**

The prototype assay measured Aβ_1–42_ with an LOD of 0.3 pg/ml and an LLOQ of 2.8 pg/ml in plasma, calibrated using an Aβ_1–42_ peptide standard from Fujirebio. Assay precision was acceptable with intra- and inter-assay coefficients of variation both being ≤10%. Dilutional linearity was demonstrated in sample diluent and immunodepleted human plasma. Analyte spike recovery ranged from 51% to 93% with a mean of 80%. This assay was able to quantify Aβ_1–42_ in all of the 84 clinical samples tested. A rapid reduction in levels of Aβ_1–42_ was detected within 1 h after drug treatment, and a dose-dependent decrease of Aβ_1–42_ levels was also observed over the time course of sample collection.

**Conclusions:**

This digital ELISA has potential utility in clinical applications for quantification of Aβ_1–42_ in plasma where high sensitivity and precision are required.

## Background

The major pathologic events associated with the development of Alzheimer’s disease (AD) are aggregation of amyloid-β (Aβ) peptides into plaques [1–5] and formation of neurofibrillary tangles from hyperphosphorylated tau protein in the brain [2, 5, 6]. Among the different neurotoxic Aβ isoforms, amyloid-β peptide 1–42 (Aβ_1–42_) is more prone to aggregation, hence constituting the predominant form in senile plaques [2, 7]. Measurement of Aβ_1–42_ levels in cerebrospinal fluid (CSF) has proved useful as an aid in early detection of AD, particularly when combined with other CSF AD biomarkers such as tau and phosphorylated tau proteins [8–15]. In contrast, plasma Aβ_1–42_ has been found to be of limited value as a diagnostic marker of AD, with contradictory reports from a variety of studies and investigators [13–18], although a recent study [19] demonstrates that Aβ_1–42_ is significantly decreased in subjects with AD. Nevertheless, plasma Aβ_1–42_ continues to be of great interest as a pharmacodynamic marker of γ-secretase (GS) and β-site amyloid precursor protein cleaving enzyme 1 (BACE1) drug target engagement in studies of candidate therapeutics [20, 21]. For example, monitoring the pharmacodynamic changes in plasma Aβ_1–42_ levels can aid in the dose optimization of GS or BACE1 [20–24]. Assays that are sensitive enough to allow accurate and precise quantification of low concentrations of Aβ_1–42_ in plasma in clinical trials of candidate Aβ-lowering therapeutics would benefit AD research efforts.

Currently, enzyme-linked immunosorbent assays (ELISA), including laboratory-developed tests and commercial kits that use different analytical platforms, have been specifically validated for measuring Aβ_1–42_ in CSF and serum or plasma from circulating peripheral blood [25–35]. However, in subjects who receive investigational Aβ-lowering drugs, plasma Aβ_1–42_ concentrations may decrease to levels precluding reliable quantification with currently available immunoassays. To effectively measure these very low Aβ_1–42_ concentrations and more reliably assess GS and BACE1 target engagement as well as the pharmacodynamic response profile, analytical methods with very low limits of quantification are required.

In the present study, a digital ELISA was developed for measuring plasma Aβ_1–42_ with improved sensitivity using single molecule array (Simoa) technology [36, 37] with antibodies 3D6 and 21F12, directed at the N- and C-termini of Aβ_1–42_, respectively. Simoa is based on the isolation of individual immunocomplexes formed on paramagnetic particles using standard ELISA reagents. Beads are subsequently loaded and sealed into an array of femtoliter-sized wells for digital measurement of signals. This ability to trap and detect single protein molecules provides unprecedented sensitivity compared with standard ELISA assays, where signal measurement usually occurs within the reacting mixture in comparatively large volumes [38–41]. The goals of this study were also to develop a digital ELISA using the same monoclonal antibodies used in previous clinical trials sponsored by Eli Lilly and Company (Indianapolis, IN, USA), evaluate the analytical performance, and demonstrate its ability to quantify Aβ_1–42_ in samples from subjects treated with a previously characterized Aβ-lowering agent using the fully automated Simoa HD-1 Analyzer (Quanterix, Lexington, MA, USA).

## Methods

### Reagents

Two monoclonal anti-Aβ_1–42_ antibodies (3D6 and 21F12) were obtained from ADx NeuroSciences (Gent, Belgium). A concentrated stock of Aβ_1–42_ peptide from the Quanterix commercial Simoa Aβ_1–42_ Kit (catalogue number 100093) was used for calibration during assay development. Additionally, Aβ_1–42_ peptide standard from the INNOTEST® β-Amyloid_(1-42)_ assay (catalogue number 51625; Fujirebio, Gent, Belgium) was used as a reference calibrator for analysis of clinical samples.

### Preparation of Simoa reagents

Capture beads were prepared by conjugating 3D6 (specific to the N-terminus of Aβ_1–42_) following standard two-step 1-ethyl-3-(3-dimethylaminopropyl)carbodiimide (EDAC) coupling chemistry. Briefly, carboxylated paramagnetic particles (Agilent Technologies, Santa Clara, CA, USA) were first washed three times with PBS plus 1% Tween 20 and twice with 50 mM 2-(*N*-morpholino)ethanesulfonic acid (MES), pH 6.2. The beads were activated with 0.5 mg/ml freshly prepared EDAC in cold 50 mM MES buffer (pH 6.2) for 30 minutes at room temperature. After activation and another immediate wash with cold MES buffer, the activated beads were conjugated with an optimized concentration (e.g., 0.5 mg/ml) of antibody in MES buffer (pH 6.2) for 2 h at room temperature. Following antibody coupling, the capture beads were washed twice with PBS plus 1% Tween 20, followed by blocking with PBS plus 1% bovine serum albumin (BSA) for 30 minutes. After blocking, the capture beads were stored in 50 mM Tris buffer with 1% BSA, pH 7.8, at 4 °C until required. The detection antibody (21F12, specific to the C-terminus of Aβ_1–42_) was exchanged into PBS using Amicon® Ultra 0.5-ml centrifugal filter devices (EMD Millipore, Billerica, MA, USA) and then reacted with EZ-link NHS-PEG4-Biotin (Thermo Fisher Scientific, Rockford, IL, USA) at 40- and 60-fold molar ratios of biotin to antibody for 30 minutes at room temperature. After biotinylation, the antibody was purified to remove excess free biotin using Amicon® Ultra 0.5-ml centrifugal filter devices and stored at 4 °C for future use.

### Digital ELISA development for detection of Aβ_1–42_

Capture beads were prepared using three different concentrations (0.3, 0.5, and 0.7 mg/ml) of 3D6 antibody during the bead conjugation process to optimize the target capture efficiency while maintaining a high level of monomericity (>80%). The detection antibody (21F12) was biotinylated at two different molar ratios, 40-fold and 60-fold, of biotin to antibody. Each of the three capture bead concentrations was then tested with both biotinylated detection antibody preparations (described above) individually using an abbreviated three-point calibration curve (0, 1, and 10 pg/ml of Aβ_1–42_ peptide added to calibration diluent). The assay performance was evaluated by comparing dose response, background level, and signal-to-background (S/B) ratios for all six conditions initially tested.

### Simoa assay (digital ELISA)

The prepared capture beads and biotinylated detection antibody detailed above were used to develop a digital ELISA for Aβ_1–42_ measurement using the Simoa technology on a fully automated HD-1 Analyzer as described elsewhere [36, 39–41]. During the first step, 100 μl of calibrators or diluted samples were mixed with the capture beads (3 × 10^6^/ml) and biotinylated detection antibody (0.1 μg/ml) for 35 minutes at room temperature, then washed three times with wash buffer containing 5× PBS and 0.1% Tween 20. After washing, Aβ_1–42_ captured on beads was enzymatically labeled by incubating it with 200 pM streptavidin-β-galactosidase for 5 minutes. Following a second wash, a solution of enzyme substrate, resorufin β-d-galactopyranoside, was added, and the capture beads were resuspended and then loaded into Simoa arrays, each containing 216,000 femtoliter-sized wells for detection. The assay was quantified by measurement of signals from bound analyte targets on the beads in units of average enzymes per bead (AEB) as previously described [36, 37, 41].

### Test sample preparation

Immunodepleted human plasma (IHP) was provided by Eli Lilly and Company. Ethylenediaminetetraacetic acid (EDTA) plasma from individual healthy donors was purchased from BioreclamationIVT (Hicksville, NY, USA). To enable precision studies, a pool of normal human EDTA plasma was prepared in-house by combining five individual healthy donor samples. The normal pool was divided into 1-ml aliquots using microcentrifuge tubes (catalogue number C-3228-1; BioExpress, Kaysville, UT, USA) and stored frozen at −80 °C. Quality control (QC) pools, designated control 1 to control 3, were also prepared by spiking Aβ_1–42_ peptide into calibration diluent at three different levels (20, 5, and 0.7 pg/ml). After preparation, QC pools were divided into 0.5-ml single-use aliquots using the microcentrifuge tubes (as above) and stored at −80 °C.

### Limit of detection and limit of quantification

To estimate the limit of detection (LOD) using optimized reagents, eight-point calibration curves were freshly prepared from a stock of Aβ_1–42_ peptide by serial dilution into calibration diluent at seven levels plus a blank (calibration diluent). Calibration curves were tested over ten runs, with each calibrator run in triplicate. The mean of AEB, SD, and coefficient of variation percentage (CV%) were calculated for each calibrator, respectively. The lower limit of quantification (LLOQ) was determined by repeated testing of the spiked IHP samples at three different levels approaching the estimated LOD of the assay.

The LLOQ for the Quanterix Aβ_42_ commercial assay was determined using the eight-point kit calibration curve. Each calibrator was tested in duplicate wells in two separate assay runs. The mean of AEB, SD, and CV% was calculated for each calibrator. The calibrator AEB values were also interpolated against the fitted four-parameter logistic (4PL) curve, and mean concentration, SD, and CV% were calculated. The LLOQ was determined by multiplying the lowest standard concentration with an interpolated CV ≤20% (and above the LOD of the assay) with the minimum required dilution (4×) of a sample.

### Intra- and inter-assay precision

Intra- and inter-assay precision were determined by testing three aliquots of the pooled normal human EDTA plasma (six replicates per aliquot) and two aliquots from controls 1–3 (in duplicate), repeated on 3 separate days. Within-run CV was calculated for the pooled plasma (*n* = 18) and with each of the three QC samples (*n* = 4) having expected values of 20 pg/ml (QC1), 5 pg/ml (QC2) and 0.7 pg/ml (QC3), respectively. Inter-assay precision was determined by averaging the results from different days (*n* = 3) and calculating the CV% for each sample.

### Dilutional linearity

Two plasma pools were investigated for dilutional linearity: one prepared from combined specimens from subjects treated with the BACE1 inhibitor LY2811376 and the other from a spiked normal human EDTA plasma pool prepared with addition of 60 pg/ml of Aβ_1–42_. For each plasma pool, parallel twofold serial dilutions were performed using sample diluent and IHP until the LLOQ was reached, respectively. The dilution-corrected concentrations of Aβ_1–42_ were calculated from the interpolated concentrations at each dilution multiplied by the corresponding dilution factors.

### Spike recovery

Four individual normal plasma samples were spiked with two levels of Aβ_1–42_ peptide, 5 and 20 pg/ml, and then diluted four-fold using sample diluent. Nonspiked samples were tested in parallel and used to calculate spike recovery.

### Clinical sample sources

Clinical samples from two phase I studies of the BACE1 inhibitor LY2886721 (Eli Lilly and Company) were used to evaluate the utility of the prototype digital ELISA in comparison with the commercial Simoa assay. In study I4O-MC-BACA, healthy volunteers received single oral doses in the range of 1–35 mg, or placebo, and in study I4O-MC-BACB, healthy volunteers received multiple oral doses of 5, 15, or 35 mg, or placebo, for 14 consecutive days. Samples selected from study I4O-MC-BACA were from subjects who received 7 mg (*n* = 3), 15 mg (*n* = 3), or 35 mg (*n* = 3) of LY2886721 or placebo (*n* = 3), collected before dose administration and 1, 6, and 12 h after dosing. Samples selected from study I4O-MC-BACB were collected before dose administration on days 1 (pretreatment baseline), 4, 8, and 12 from subjects who received 5 mg (*n* = 3), 15 mg (*n* = 3), or 35 mg (*n* = 3) of LY2886721. Additionally, samples from part B of a phase I clinical trial (I3J-MC-LACE) of the BACE1 inhibitor LY2811376 (Eli Lilly and Company), where healthy volunteers received single oral doses of 30 mg, 90 mg, or placebo [22], were combined to prepare low-concentration pools for assay precision testing.

All clinical studies were conducted in compliance with the revised (1996) Helsinki declaration of 1975, and all enrolled subjects provided informed consent for treatment and use of samples for research. The protocols permitted collection of plasma specimens before and during treatment for exploratory biomarker measurements. Blood specimens were collected into 6-ml plastic K_2_EDTA-coated BD Vacutainer tubes (catalogue number 367863; BD Biosciences, San Jose, CA, USA). After separation, plasma was divided into 0.5-ml aliquots and stored frozen at less than or equal to −70 °C in 2-ml screw-topped polypropylene vials (catalogue number 72.694.056; Sarstedt, Nümbrecht, Germany).

### Measurement of Aβ_1–42_ in human plasma pools and clinical samples

Plasma samples stored at −80 °C were first thawed at room temperature, then mixed by brief vortexing and centrifuged at 20,000 × *g* for 3 minutes to pellet any particulates. The supernatant was removed, then diluted four-fold with sample diluent and analyzed using a digital ELISA. Clinical samples were analyzed in batches by subject. Two 8-point calibration curves, one prepared using the Aβ_1–42_ peptide from Quanterix and the other with material from Fujirebio, were included in each batch, in addition to one aliquot from two QC pools (QC1 and QC3) already described, to verify run validity. All calibrators, QC, and clinical samples were tested in duplicate, with a single result reported.

### Simoa comparison

All clinical samples were analyzed in parallel using the Simoa Aβ_1–42_ assay described above and the commercial Simoa kit from Quanterix that employs different antibody reagents for Aβ_1–42_ capture (specific to the N-terminus of Aβ_1–42_) and detection (specific to the C-terminus of Aβ_1–42_). The Aβ_1–42_ concentrations quantified from both assays were compared for all 84 samples tested. Additionally, on the basis of the results of clinical sample analysis, theoretical projections were made of each assay’s ability to quantify decreasing concentrations of Aβ_1–42_ resulting from treatment with Aβ-lowering therapeutics.

### Aβ_1–42_ calibration standard comparison

As a result of an observed difference in response of the calibrators, Aβ_1–42_ peptides from the Quanterix commercial Simoa Aβ_1–42_ Kit (catalogue number 100093) and the INNOTEST® β-Amyloid_(1–42)_ assay from Fujirebio (catalogue number 51625) were compared independently of the Simoa assay. Because of carrier proteins present in the standards, it was not possible to use amino acid analysis (AAA) to directly confirm concentrations. Therefore, each standard was run in an acid urea gel [42] under totally denatured conditions and compared with an Eli Lilly reference standard prepared in formic acid and subjected to AAA. Briefly, the Lilly reference standard was diluted in formic acid to prepare a standard curve, and amounts of 1, 0.5, 0.25, 0.125, and 0.0625 ng were loaded into wells on the acid urea gel. Additionally, the Quanterix standard, the INNOTEST® standard, and another Lilly substock of corporate reference standard were each diluted to achieve 0.25 ng/well on the basis of their stated concentrations. Three sets of duplicates were made to run on two gels, thereby achieving individually diluted standard samples as triplicates (duplicates for the Eli Lilly substock because of space limitations on the gel). A Western blot using 3D6 antibody detection was employed to detect Aβ_1–42_ peptide bands. A quadratic equation of the AAA standard curve was used to calculate the actual value for each of the Quanterix, INNOTEST®, and Lilly substock standard samples.

### Data analysis

A 4PL fit was used to calibrate the Quanterix commercial Aβ_1–42_ kit assay according to the package insert, whereas a cubic calibration fit was used to quantify Aβ_1–42_ with the prototype assay reported herein. For the prototype Aβ_1–42_ assay, a cubic calibration regression model provided better fitting accuracy than a 4PL, particularly for the upper end of the calibration range. Mean AEB, SD, and CV% were calculated for all measured Aβ_1–42_ concentrations.

## Results

### Assay calibration

A summary of the calibrator performance in each of the prototype assay runs is presented in Table 1. All runs included Quanterix calibrators. Calibration performance with the Quanterix standard was acceptable over the range of 0.206–50 pg/ml with back-calculated relative error between −2.5% and +12.4%. Inter-assay CV varied from 0.5% to 19% over the same calibration range. The measurement range of the assay in plasma using the Quanterix standard, based on a four-fold dilution, was 0.824–200 pg/ml.

**Table 1:** Prototype assay calibration performance summary using Quanterix standard for assay evaluation and clinical test batches

	Concentration^a^ (pg/ml)							
Theoretical concentration	0.023	0.069	0.206	0.617	1.85	5.6	16.7	50.0
Run 1		0.096	0.199	0.616	1.85	5.56	16.9	49.9
Run 2	−0.011	0.066	0.224	0.614	1.77	5.67	17.2	49.7
Run 3	−0.032	0.135	0.281	0.613	1.78	5.56	17.4	49.5
Run 4	−0.012	0.080	0.201	0.615	1.87	5.48	16.9	49.9
Run 5	−0.074	0.103	0.218	0.602	1.85	5.54	16.9	49.9
Run 6	0.061	0.088	0.194	0.620	1.85	5.48	17.0	49.8
Run 7		0.066	0.202	0.627	1.82	5.56	16.8	49.9
Run 8		0.234	0.323	0.525	2.11	5.23	17.5	49.8
Run 9		0.214	0.240	0.583	1.82	5.43	17.8	49.2
N (total number of runs)	5	9	9	9	9	9	9	9
Inter-assay mean	−0.014	0.120	0.231	0.602	1.86	5.5	17.2	49.7
SD	0.049	0.063	0.044	0.031	0.099	0.121	0.350	0.237
CV%	−362	52.1	19.0	5.2	5.3	2.2	2.0	0.5
Inter-assay RE, %	−159	74.2	12.4	−2.5	0.4	−1.8	2.7	−0.5

*CV%* Coefficient of variation percentage, *RE* Relative error

In runs 6–8, calibration curves prepared using both Quanterix and Fujirebio standards were included

^a^Concentrations were interpolated using the calibrator average enzymes per bead values against the fitted cubic curves

Runs 6–8 included a calibration series prepared with standard peptide from an INNOTEST® commercial assay kit. These results are presented in Table 2. An acceptable performance with the INNOTEST® calibrator was obtained over the range of 0.617–50 pg/ml, which translates to 2.47–200 pg/ml of plasma when a four-fold dilution is applied. Summaries of AEB for each of the calibration curves from the prototype assay runs are presented in Tables 3 and 4 for both calibrators.

**Table 2:** Prototype assay calibration performance summary using Fujirebio standard for assay evaluation and clinical test batches

	Concentration^a^ (pg/ml)							
Theoretical concentration	0.023	0.069	0.206	0.617	1.85	5.6	16.7	50.0
Run 6	−0.006	0.097	0.262	0.584	1.83	5.65	16.6	50.1
Run 7		0.112	0.365	0.514	1.94	5.36	17.1	49.8
Run 8		0.260	0.283	0.388	1.98	5.79	16.0	50.5
N (total number of runs)	1	3	3	3	3	3	3	3
Inter-assay mean	−0.006	0.156	0.303	0.495	1.92	5.6	16.6	50.1
SD		0.090	0.054	0.100	0.077	0.220	0.508	0.365
CV%		57.9	17.9	20.1	4.0	3.9	3.1	0.7
Inter-assay RE, %	−125	128	47.5	−19.8	3.6	0.8	−0.7	0.3

*CV%* Coefficient of variation percentage, *RE* Relative error

In runs 6–8, calibration curves prepared using both Quanterix and Fujirebio standards were included

^a^Concentrations were interpolated using the calibrator average enzymes per bead values against the fitted cubic curves

**Table 3:** Summary of average enzymes per bead for prototype assay calibration using Quanterix standard for assay evaluation and clinical test batches

	AEB							
Theoretical concentration, pg/ml	0.023	0.069	0.206	0.617	1.85	5.6	16.7	50.0
Run 1	0.006	0.008	0.010	0.023	0.112	0.794	5.951	27.615
Run 2	0.005	0.006	0.008	0.018	0.086	0.692	5.214	23.725
Run 3	0.005	0.007	0.009	0.018	0.089	0.703	5.774	29.431
Run 4	0.006	0.006	0.008	0.020	0.102	0.720	5.665	28.302
Run 5	0.005	0.006	0.008	0.018	0.096	0.720	5.603	28.131
Run 6	0.006	0.007	0.009	0.019	0.089	0.624	5.082	27.607
Run 7	0.011	0.012	0.014	0.025	0.091	0.618	4.674	25.809
Run 8	0.007	0.009	0.011	0.015	0.107	0.580	5.454	24.691
Run 9	0.007	0.009	0.010	0.018	0.084	0.608	5.409	24.587
N (total number of runs)	9	9	9	9	9	9	9	9
Inter-assay mean	0.006	0.008	0.010	0.019	0.095	0.673	5.425	26.655
SD	0.00	0.00	0.00	0.00	0.01	0.07	0.39	2.00
CV%	26.4	24.8	19.1	15.4	10.4	10.3	7.2	7.5

*AEB* Average enzyme per bead, *CV%* Coefficient of variation percentage

In runs 6–8, calibration curves prepared using both Quanterix and Fujirebio standards were included

**Table 4:** Summary of average enzymes per bead for prototype assay calibration using Fujirebio standard for assay evaluation and clinical test batches

	AEB							
Theoretical concentration, pg/ml	0.023	0.069	0.206	0.617	1.85	5.6	16.7	50.0
Run 6	0.006	0.007	0.008	0.012	0.031	0.164	1.105	8.401
Run 7	0.011	0.012	0.014	0.015	0.036	0.142	1.083	8.115
Run 8	0.011	0.012	0.013	0.013	0.033	0.149	0.931	8.084
N (total number of runs)	3	3	3	3	3	3	3	3
Inter-assay mean	0.009	0.010	0.012	0.013	0.033	0.151	1.040	8.200
SD	0.003	0.003	0.003	0.002	0.002	0.011	0.095	0.175
CV%	28.8	29.1	25.0	13.8	6.6	7.3	9.1	2.1

*AEB* Average enzyme per bead, *CV%* Coefficient of variation percentage

In runs 6–8, calibration curves prepared using both Quanterix and Fujirebio standards were included

**Fig. 1:**
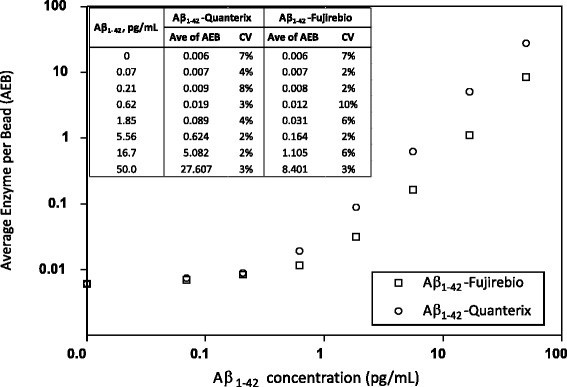
Representative dose-response curves using the amyloid-β 1–42 peptides (Aβ_1–42_) from Quanterix and Fujirebio. For each calibration curve, average enzymes per bead (AEB) (*n* = 3) and coefficient of variation (CV) of each calibration level are shown in the embedded table. The signals from the Quanterix Aβ_1–42_ peptide were approximately threefold higher than those from the Fujirebio Aβ_1–42_ peptide

For comparison, calibration curves were prepared using Aβ_1–42_ peptide standards from Quanterix and Fujirebio to evaluate the sensitivity of this assay. Figure 1 shows the representative calibration curves determined using both Aβ_1–42_ peptides. The signals from the Quanterix Aβ_1–42_ peptide were approximately threefold higher than those from the Fujirebio Aβ_1–42_ peptide.

### Aβ_1–42_ calibration standard comparison

**Fig. 2:**
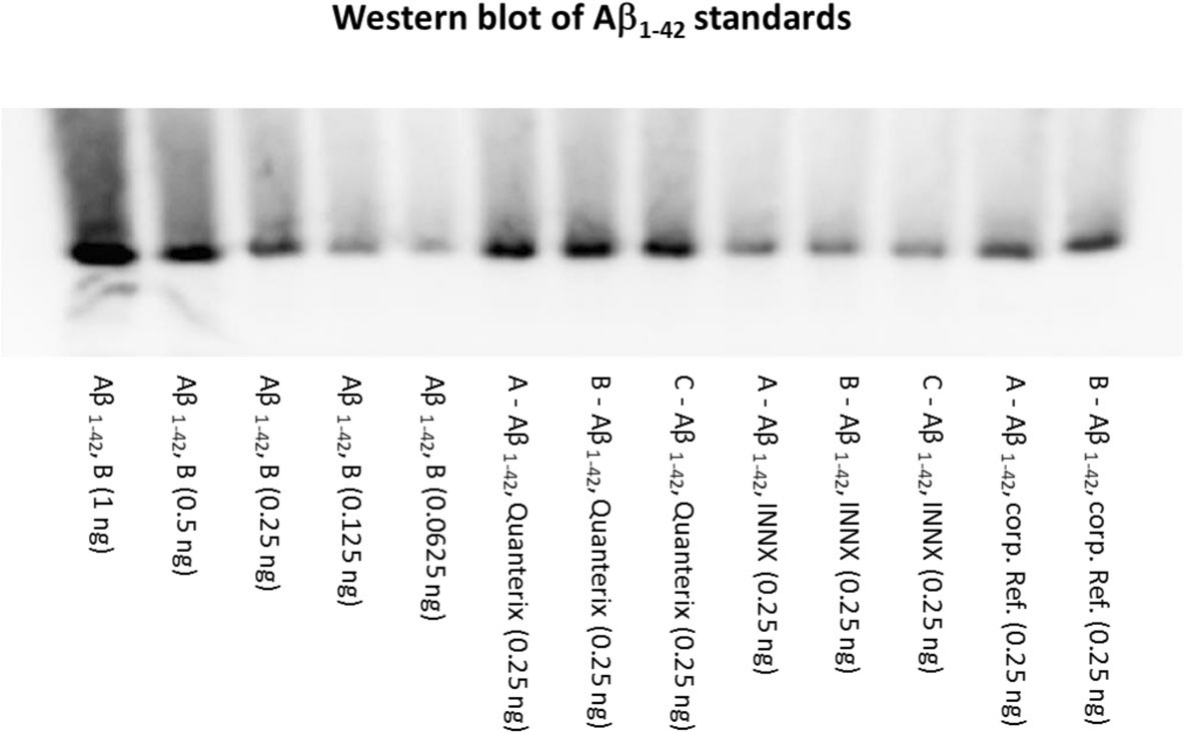
Western blot of amyloid-β 1–42 peptide (Aβ_1–42_) peptide standards after 3D6 antibody detection following quantitative acid gel electrophoresis. *Lanes 1–5* (*left* to *right*),_1–42_ reference standard from Lilly with amounts of 1, 0.5, 0.25, 0.125, and 0.0625 ng, respectively, loaded into each well. *Lanes 6–8* show three replicates of the diluted Quanterix Aβ_1–42_ standard. *Lanes 9–11* show three replicates of the diluted INNOTEST® Aβ_1–42_ standard. *Lanes 12* and *13* show two replicates of another Lilly substock of Aβ_1–42_ reference standard. From *lane 6* to *lane 13*, 0.25 ng of each Aβ_1–42_ peptide was loaded per well, based upon their stated concentrations

A 3D6 Western blot of Aβ_1–42_ standards is shown in Fig. 2, and the measured concentrations of the two commercial standards interpolated from the Lilly standard calibration curve are presented in Table 5. The measured amount of Aβ_1–42_ in the Lilly peptide standard was close to the target value of 0.25 ng, whereas the Quanterix and Fujirebio standards were considerably higher (+47%) and lower (−51%), respectively.

**Table 5:** Estimated amyloid-β 1–42 peptide content of nominal 0.25-ng masses of calibration standard

Calibration standard	Theoretical amount loaded (ng)	Mean amount measured (ng)	Relative error (%)	SEM
Quanterix^a^	0.250	0.367	+47	8.1
Fujirebio^a^	0.250	0.123	−51	7.3
Lilly^b^	0.250	0.230	−8	15.0

^a^ *n* = 3

^b^ *n* = 2

### Limit of detection and lower limit of quantification

The LOD and LLOQ results are presented in Table 6. LOD is defined as an interpolated Aβ_1–42_ level derived from a measured signal equivalent to the assay background from the buffer blank plus 2.5 times the SD. The calculated LOD for the prototype assay using the Quanterix peptide standard from ten calibration curves ranged from 0.09 to 0.15 pg/ml with an average of 0.12 pg/ml, using a cubic fit regression model. The LLOQ was determined by repeated testing of the spiked IHP samples. The mean concentrations of Aβ_1–42_ quantified from these samples (based on a Quanterix Aβ_1–42_ peptide calibration curve) after four-fold dilution were 0.3, 0.4, and 0.6 pg/ml with CV% of 8%, 7%, and 7%, respectively. Defining the LLOQ as the lowest Aβ_1–42_ concentration that can be reliably quantified from plasma samples with a CV% ≤20% from repeated measurements, it was determined to be 1.2 pg/ml of plasma, after accounting for the four-fold preassay dilution.

**Table 6:** Comparison of sensitivity using two different amyloid-β 1–42 peptides as calibrators

Aβ_1–42_ peptide calibrator	LOD (pg/ml)	LLOQ (pg/ml)^a^
Quanterix	0.12	1.2
Fujirebio	0.31	2.8

*Aβ* amyloid-β, *LLOQ* Lower limit of quantification, *LOD* Limit of detection

^a^Aβ_1–42_ levels in neat plasma, accounting for four-fold preassay sample dilution

When applied to the INNOTEST® Aβ_1–42_ peptide standard, the LOD calculated from three calibration curves was 0.31 pg/ml, and the LLOQ determined from measurement of spiked IHP was 2.8 pg/ml of plasma.

### Intra- and inter-assay precision

The overall results from intra- and inter-assay precision are summarized in Table 7. Intra-assay CV% varied between 0.1% and 8%, and inter-assay CV% varied between 2% and 8%.

**Table 7:** Summaries of intra- and inter-assay precision for repeated testing of three quality control samples and one plasma pool

	Aβ_1–42_ (pg/ml)			
Sample	QC1	QC2	QC3	Plasma pool^a^
Intra-assay means (*n* = 4)	20.5	4.89	0.696	5.32
	20.0	4.87	0.628	5.00
	19.6	4.76	0.745	5.32
Intra-assay CV%	2	2	1	1
	4	1	7	1
	0.4	0.1	8	3
Inter-assay means (*n* = 3)	20.0	4.84	0.689	5.20
Inter-assay CV%	2	2	8	4

*Aβ* amyloid-β, *CV%* Coefficient of variation percentage, *QC* Quality control

^a^ *n* = 18 for plasma pool; the reported Aβ_1–42_ levels in plasma pool were corrected for four-fold sample dilution

### Dilutional linearity

**Fig. 3:**
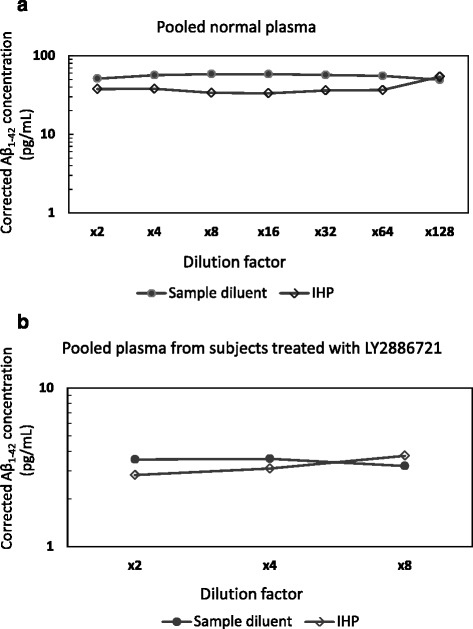
The effect of sample dilution on quantification of amyloid-β (Aβ_1–42_) in plasma. Pooled ethylenediaminetetraacetic acid plasma spiked with 60 pg/ml of Aβ_1–42_ peptide (**a**) and pooled samples from subjects treated with LY2886721 (**b**) were initially diluted twofold, followed by serial dilutions with sample diluent or immunodepleted human plasma (IHP). Dilutional linearity was observed using both types of diluent, ranging from 91% to 115% with sample diluent buffer (from 2- to 128-fold dilution) and from 89% to 120% with IHP (from 2- to 64-fold dilution)

Figure 3 highlights the linearity of Aβ_1–42_ measurement in a spiked pool from normal human EDTA plasma (Fig. 3a) and a pool prepared from drug-treated subjects (Fig. 3b) diluted with both sample diluent and IHP, respectively. Any dilutions that resulted in quantified Aβ_1–42_ levels less than the LLOQ were excluded from analysis. For example, only three dilutions (two- to eightfold) are displayed for the pooled subjects treated with LY2811376, because starting concentrations in these drug-treated subjects were already very low. All corrected reportable values were within 20% of the nominal concentration obtained using a four-fold dilution. In addition, the CV% of the replicate determinations at each dilution within the assay range were ≤20% for two- to eightfold dilutions. As shown in Fig. 3, dilutional linearity was observed for both types of pooled plasma samples with each diluent investigated. Linearity ranged from 91% to 115% when sample diluent was used (from 2- to 128-fold dilution) and 89% to 120% when using IHP as the diluent (from 2- to 64-fold dilution).

### Analyte spike recovery

The recovery was calculated by subtracting the measured Aβ_1–42_ concentration of the nonspiked sample from the measured concentration of the spiked sample and dividing the result by the concentration spiked into each sample, reported as a percentage of the added spike. Recovery of spiked Aβ_1–42_ from EDTA plasma was dependent on concentration and ranged from 51% to 93%, and it also varied between samples.

### Measurement of Aβ_1–42_ in clinical samples

**Fig. 4:**
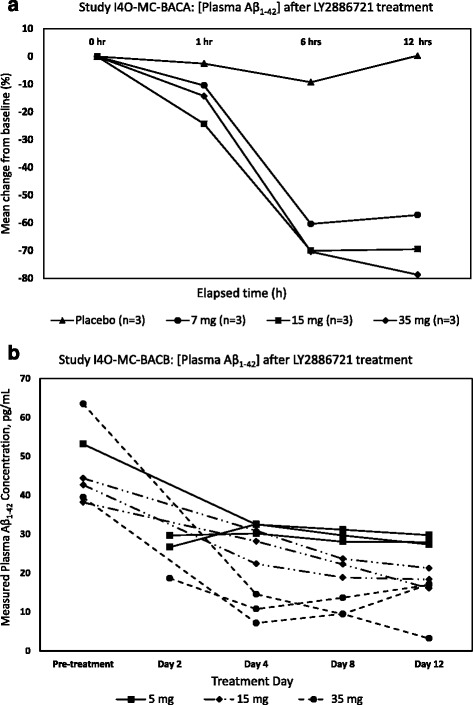
Changes in plasma amyloid-β 1–42 peptide (Aβ_1–42_) concentration in subjects who received oral doses of the β-site amyloid precursor protein cleaving enzyme 1inhibitor LY2886721 in separate clinical studies. **a** Mean percentage change in Aβ_1–42_ concentration from baseline values for subjects treated with single doses of LY2886721 in study I4O-MC-BACA. **b** Plasma Aβ_1–42_ concentration for individual subjects treated with 14 consecutive once-daily doses of LY2886721 in study I4O-MC-BACB. Samples from both studies were quantified with Fujirebio Aβ_1–42_ peptide standard

The measured Aβ_1–42_ concentrations in clinical specimens from subjects who received doses of LY2886721 are presented in Tables 8 and 9. For comparison, the Aβ_1–42_ concentrations in each sample were quantified using calibration curves generated with Quanterix and Fujirebio Aβ_1–42_ standard peptides. For study I4O-MC-BACA, the change in Aβ_1–42_ concentration compared with the baseline (predose) value was calculated for each subsequent time point over the course of the study period (Fig. 4a). For study I4O-MC-BACB, predose treatment values were not available for two subjects from the 5-mg treatment group (subjects R and S) and one subject from the 35-mg treatment group (subject Q); consequently, no change from baseline calculations were made. Instead, Fig. 4b shows measured concentrations of Aβ_1–42_ for each subject in study I4O-MC-BACB with increasing duration of treatment for each dose group. Measured concentrations and percentage changes shown in Fig. 4 were all determined using the Fujirebio Aβ_1–42_ peptide standard. Regardless of the calibrator used, the percentage change in Aβ_1–42_ concentration for the samples and calculated maximum measurable percentage changes were similar (Table 10). However, there were differences in the percentage reduction between the newly developed assay (79%) and the Quanterix commercial kit assay (64%), as listed in Table 10. Two QC samples (QC1 and QC3) measured during the clinical sample analysis were within the expected ranges (Table 11).

**Table 8:** Summary of measured amyloid-β 1–42 peptide concentrations for samples from study I4O-MC-BACA (*n* = 48) using both Quanterix and Fujirebio amyloid-β 1–42 peptides for calibration

		Aβ_1–42_ (pg/ml)							
		Quanterix Aβ_1–42_ peptide calibration				Fujirebio Aβ_1–42_ peptide calibration			
Subject	Dose	0 h	1 h	6 h	12 h	0 h	1 h	6 h	12 h
A	Placebo	16.8	16.9	17.0	18.1	39.7	39.9	40.0	42.9
D	Placebo	14.7	13.3	10.9	12.0	34.4	31.1	25.1	27.8
G	Placebo	18.6	18.8	18.3	20.8	43.9	44.6	43.3	49.4
B	7 mg	18.7	12.2	8.03	8.25	44.3	28.3	17.9	18.5
C	7 mg	21.0	19.6	7.64	6.70	49.9	46.4	17.0	14.6
E	7 mg	20.4	22.8	9.48	12.0	48.4	54.2	21.5	27.9
A	15 mg	16.9	13.1	5.65	6.61	39.8	30.6	11.9	14.3
F	15 mg	17.4	14.4	5.99	5.74	41.1	33.7	12.8	12.1
K	15 mg	20.5	14.2	6.51	5.95	48.8	33.3	14.1	12.7
H	35 mg	21.7	16.8	4.80	4.88	51.5	39.7	9.74	9.94
I	35 mg	18.6	15.5	7.43	5.27	44.1	36.4	16.4	11.0
J	35 mg	19.5	19.1	6.92	4.56	46.3	45.2	15.1	9.12

*Aβ* amyloid-β

**Table 9:** Summary of measured amyloid-β 1–42 peptide concentrations for samples from study I4O-MC-BACB (*n* = 36) using both Quanterix and Fujirebio amyloid-β 1–42 peptides for calibration

		Aβ_1–42_ (pg/ml)									
		Quanterix Aβ_1–42_ peptide calibration					Fujirebio Aβ_1–42_ peptide calibration				
Subject	Dose	Predose	Day 2	Day 4	Day 8	Day 12	Predose	Day 2	Day 4	Day 8	Day 12
R	5 mg		11.9	14.5	13.2	12.2		26.7	32.6	29.7	27.4
S	5 mg		13.2	13.4	12.5	12.5		29.7	30.2	28.1	28.0
T	5 mg	23.6		14.4	13.8	13.2	53.2		32.5	31.2	29.8
L	15 mg	19.0		9.98	8.43	8.24	42.7		22.4	18.9	18.4
M	15 mg	16.9		12.5	9.96	7.22	38.2		28.2	22.3	16.1
N	15 mg	19.7		13.7	10.5	9.52	44.4		30.9	23.7	21.3
O	35 mg	17.6		3.33	4.35	1.65	39.5		7.19	9.54	3.22
P	35 mg	28.3		6.58	4.33	7.70	63.5		14.6	9.48	17.2
Q	35 mg		8.37	4.88	6.15	7.61		18.7	10.8	13.7	17.0

*Aβ* amyloid-β

**Table 10:** Comparison of prototype digital enzyme-linked immunosorbent assay (Simoa) amyloid-β 1–42 peptide assay with the Quanterix commercial Simoa assay in the context of use as a pharmacodynamic marker in β-site amyloid precursor protein cleaving enzyme 1 inhibitor clinical trials

ELISA	LLOQ (pg/ml; ±20%)	Mean baseline (pg/ml) (SD)	Maximum percentage reduction (SD)	Maximum quantifiable percentage reduction (±20%)
Quanterix commercial Aβ_1–42_ assay performance				
Aβ_1–42_ peptide calibrator				
Quanterix	0.274 (0.220–0.329)^a^	17.1 (2.16)^b^	64 (5)^c^	98 (96.5–98.7)
Fujirebio	0.824 (0.659–0.989)^a^	39.6 (4.93)^b^	64 (5)^c^	98 (95.4–98.3)
Prototype Simoa Aβ_1–42_ assay performance				
Aβ_1–42_ peptide calibrator				
Quanterix	1.2 (0.96–1.44)	18.7 (2.04)^d^	75 (3)^e^	94 (92.3–94.9)
Fujirebio	2.8 (2.24–3.36)	44.35 (4.99)^d^	79 (3)^e^	94 (92.4–94.9)

*Aβ* amyloid-β, *ELISA* Enzyme-linked immunosorbent assay, *LLOQ* Lower limit of quantification

*Note:* Quanterix assay data show the maximum reduction of signal observed in I4O-MC-BACA and the assay’s maximum quantifiable percentage reduction limit for the Quanterix commercial assay. Prototype Simoa assay data show results for the same parameters when using the newly developed Simoa assay

^a^LLOQ based on lowest standard with replicate performance <20% coefficient of variation

^b^Baseline values are calculated from study I4O-MC-BACA (not shown)

^c^Maximum percentage reduction calculated from 35-mg dose group of study I4O-MC-BACA (not shown)

^d^Mean baseline values are calculated from study I4O-MC-BACA (Table 3)

^e^Maximum percentage reduction calculated from 35-mg dose group of study I4O-MC-BACA (Table 5)

**Table 11:** Summaries of plasma amyloid-β 1–42 peptide assay measured from quality control samples (QC1 and QC3) during clinical sample analysis

	Measured Aβ_1–42_ (pg/ml)			
	Run 1	Run 2	Run 3	Expected range of Aβ_1–42_ (pg/ml)^a^
QC1	21.6	21.4	19.4	20 ± 2
QC3	0.748	0.681	0.642	0.689 ± 0.069

*Aβ* amyloid-β, *QC* Quality control

^a^Expected range was within ±10% of the averaged Aβ_1–42_ concentration measured from precision study

### Simoa assay comparison

**Fig. 5:**
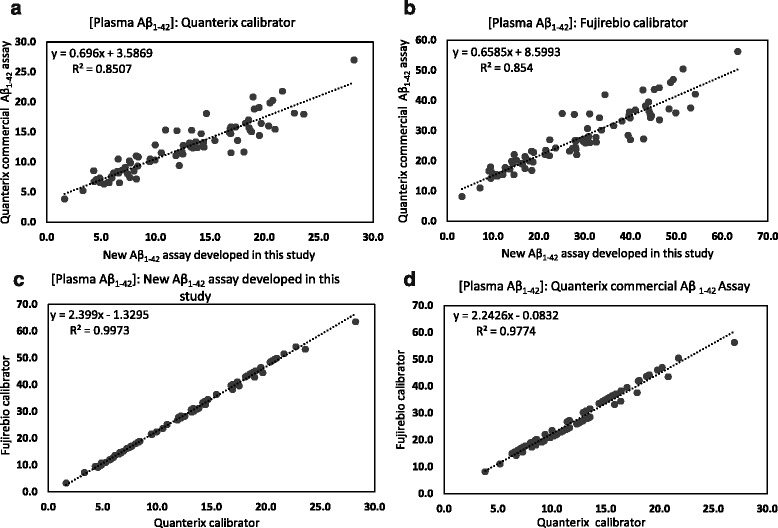
Correlations of measured amyloid-β 1–42 peptide (Aβ_1–42_) concentrations in clinical samples when using two different Aβ_1–42_ assays (assay developed herein or Quanterix commercial kit assay) for sample analysis (**a** and **b**) and when using two different Aβ_1–42_ peptide standards (Quanterix or Fujirebio) for calibration (**c** and **d**). Results from both assays were correlated (*r* ^2^ = 0.85), regardless of the calibrator used. Similarly, sample measurements with each calibrator were correlated within each assay (*r* ^2^ > 0.9). A total of 84 samples were tested

A comparison of clinical sample results between the new Simoa Aβ_1–42_ assay reported here and the commercial Quanterix assay are presented in Fig. 5. Results from both assays correlated well (*r* ^2^ = 0.85) (Fig. 5a and b). A total of 84 samples were tested using both assays, and each assay was calibrated using both available peptide sources. The correlations between results quantified using the different Aβ_1–42_ peptide standards were very high within each assay, as shown in Fig. 5c (*r* ^2^ = 0.997) and Fig. 5d (*r* ^2^ = 0.977).

## Discussion

Simoa technology uses an array of femtoliter-sized reaction chambers that is designed to capture and detect single peptides, resulting in a strong, rapidly developed fluorescent signal from a very small mass of analyte. The aim of this study was to develop and evaluate a Simoa assay for Aβ_1–42_ with the same amyloid-β-specific antibodies (3D6 and 21F12) used as reagents in previous studies for measurements in human CSF and plasma following treatment with BACE inhibitors [24, 32, 35]. The analytical sensitivity of existing assays available was only just sufficient to quantify the extremely low plasma levels of Aβ_1–42_ resulting from treatment [32], suggesting a need for improved assays to support development of more potent BACE inhibitors in the future. The performance characteristics of the prototype Simoa assay were evaluated, and the assay’s capability for quantification of Aβ_1–42_ at reduced levels in human plasma for pharmacodynamic evaluations related to the development of AD therapeutic targets was demonstrated.

To achieve better sensitivity using antibodies 3D6 and 21F12, Simoa reagents, assay buffers, and assay conditions were optimized. High-performance Simoa immunoassays, like conventional immunoassays, benefit from the same desirable characteristics, such as low background, high S/B ratio (robust dose-response slope), and a dynamic range suitable for the assay’s intended use. Of the six conditions evaluated, capture beads coated with 0.5 mg/ml 3D6 antibody and 21F12 antibody biotinylated at 40-fold were determined to yield the most favorable assay performance with the best S/B ratio. Hence, these conditions were used to evaluate the Simoa Aβ_1–42_ assay for analytical sensitivity and other assay performance characteristics.

For immunoassays, the choice of calibration regression models is essential to achieve accurate quantification for sample measurement. Although a 4PL fit is the most widely used regression model for immunoassays, a cubic fit was found to yield an overall better calibration accuracy for the prototype Simoa assay, with relative errors from back-fitted concentrations compared with the corresponding theoretical values typically <5% for any calibrators greater than the LLOQ and a fit coefficient value (*r* ^2^) >0.99. Conversely, the relative errors were generally >20% across the calibration range with *r* ^2^ <0.5 using a 4PL regression model. Consequently, a cubic calibration fit was chosen for sample quantification using the prototype Simoa assay.

In addition to low picogram-per-milliliter sensitivity, a major advantage of this Simoa assay is the linear Aβ_1–42_ quantification observed when diluting plasma using sample diluent in standard tests or IHP in proportional linearity tests. Nonlinear assays restrict sample measurements to the calibrated range [32, 43]. In contrast, this assay has shown robust dilutional linearity in the range of 2- to 128-fold in measurements from a normal plasma pool spiked with Aβ_1–42_ peptide. The dilutional linearity highlighted here allows samples that are well above the calibration range to be diluted into range without affecting the accuracy of measurement, thus expanding the application to clinical samples that have a much wider range of Aβ_1–42_ levels. Additionally, sample dilution minimizes the matrix effects that are known to hinder accurate Aβ_1–42_ measurement in plasma samples using ELISA [32, 33, 43, 44].

Reproducibility of the assay was evaluated using both pooled normal plasma and QC samples prepared in calibration diluent at three different levels spanning the assay calibrated range. Both intra- and inter-assay CV% were <10% at all concentrations tested over a period of 3 days, demonstrating high assay precision and reproducibility for Aβ_1–42_ measurements.

The results of this study have also highlighted an unexpected major difference in the concentration value assignments of two Aβ_1–42_ peptide standards used to calibrate the assays. The signal responses from the Quanterix Aβ_1–42_ antigen were found to be significantly higher than those from the Fujirebio standard, although they both had the same assigned concentrations (Fig. 1). The reported Aβ_1–42_ concentrations from the Fujirebio peptide calibration curve are approximately 2.4-fold higher across the range of samples tested (slope = 2.399, *R* ^2^ = 0.9973; Fig. 5c) compared with the Quanterix calibration. For the predose samples, the Fujirebio-based Aβ_1–42_ concentrations are typically in the range of 35–60 pg/ml, in line with previously reported results obtained using Fujirebio assays [33].

To gain a greater understanding of the differences between the two calibration standards used in the present methods study, the Aβ_1–42_ peptide content was determined using a quantitative acid gel electrophoretic procedure that is run routinely at Lilly Research Laboratories (Indianapolis, IN, USA). Because of carrier proteins present in the standards, it was not possible to use AAA to directly confirm concentrations of each standard. Therefore, each standard was run on a totally denaturing gel system against an AAA-verified standard. The results showed that the Quanterix standard contained threefold more Aβ_1–42_ peptide than the Fujirebio standard at the same assigned concentration. These disparate Aβ_1–42_ peptide standard contents immediately explain the apparent difference in measured Aβ_1–42_ concentrations in clinical samples, depending on the selected calibrator, as well as the difference in calibrator response observed during prototype assay development. Further investigation of the differences between these two standards, involving empirical chemical tests, may be warranted. Standardization of antigens for calibration has been a general challenge with different immunoassays in the absence of a universal reference standard. Recent progress toward availability of a globally recognized standard for Aβ_1–42_ will facilitate comparisons of results from various assay platforms and improve clinical interpretation [45, 46].

To demonstrate the potential clinical utility of this assay for sensitive and reliable quantification of Aβ_1–42_, a total of 84 plasma samples from subjects treated with the BACE1 inhibitor LY2886721 in two clinical trials were tested. This Simoa assay was able to accurately quantify Aβ_1–42_ in all plasma samples tested, demonstrating its capability to support studies of Aβ-lowering therapeutics for AD. Similar to previous findings, oral administration of LY2886721 produced rapid and sustained reductions in plasma Aβ_1–42_ levels [24, 32]. Compared with placebo treatment, reductions of Aβ_1–42_ concentrations were measureable within 1 h, and remained relatively unchanged after 6 h, following single oral doses of LY2886721 in study I4O-MC-BACA. In study I4O-MC-BACB, where subjects received multiple oral doses of 5 mg or 15 mg of LY2886721, continuous reductions in Aβ_1–42_ levels were observed over 12 days. However, in the same study, plasma Aβ_1–42_ reached a nadir after day 4, and further decreases in concentration were relatively minor for those treated with 35 mg of LY2886721. In both studies, a clear, dose-dependent effect of lowering plasma Aβ_1–42_ concentrations was seen (Fig. 4). The assay developed herein was also evaluated in parallel with the Quanterix commercial Aβ_1–42_ assay for utility in clinical sample analysis. Although the Quanterix Aβ_1–42_ assay uses different antibodies from those used to develop the present assay, it exhibits similar analytical sensitivity, and results from the two assays were correlated (*r* ^2^ = 0.85).

## Conclusions

The digital ELISA developed in this study provided sensitive and precise measurement of plasma Aβ_1–42_ in clinical samples treated with the BACE1 inhibitor LY2886721. The improved assay sensitivity enabled Aβ_1–42_ to be reliably quantified even at low picogram-per-milliliter levels. Linearity of measurement with increasing sample dilution was demonstrated, allowing clinical samples with a much larger range of Aβ_1–42_ concentrations to be tested without impacting measurement accuracy. Moreover, the digital ELISA was performed with full automation on the HD-1 Analyzer, with a fast assay turnaround time of <65 minutes from sampling to results. With the performance demonstrated in this evaluation study, this Simoa Aβ_1–42_ assay is well-suited for use in studies that involve AD therapeutic agents aimed at lowering plasma Aβ concentrations.

## Abbreviations

AAA: Amino acid analysis
Aβ: Amyloid-β
AD: Alzheimer’s disease
AEB: Average enzymes per bead
BACE1: β-Site amyloid precursor protein cleaving enzyme 1
BSA: Bovine serum albumin
CSF: Cerebrospinal fluid
CV%: Coefficient of variation percentage
EDAC: 1-Ethyl-3-(3-dimethylaminopropyl)carbodiimide
EDTA: Ethylenediaminetetraacetic acid
ELISA: Enzyme-linked immunosorbent assay
GS: γ-Secretase
IHP: Immunodepleted human plasma
LLOQ: Lower limit of quantification
LOD: Limit of detection
MES: 2-(*N*-morpholino)ethanesulfonic acid
4PL: Four-parameter logistic
QC: Quality control
RE: Relative error
S/B: Signal-to-background ratio
Simoa: Single molecule array
